# Systemic central venous oxygen saturation is associated with clot strength during traumatic hemorrhagic shock: A preclinical observational model

**DOI:** 10.1186/1757-7241-18-64

**Published:** 2010-12-07

**Authors:** Nathan J White, Erika J Martin, Yongyun Shin, Donald F Brophy, Robert F Diegelmann, Kevin R Ward

**Affiliations:** 1 Reanimation Engineering Science Center, Virginia Commonwealth University, (1200 East Broad Street) Richmond, Virginia (23298) USA; 2 Coagulation Advancement Laboratory, Department of Pharmacotherapy and Outcomes Science, Virginia Commonwealth University, (1112 E. Clay Street) Richmond, Virginia (23298) USA; 3 Department of Emergency Medicine, Virginia Commonwealth University, (1200 Marshall Avenue) Richmond, Virginia (23223) USA; 4 Department of Pharmacotherapy and Outcomes Science, Virginia Commonwealth University, (410 North 12th Street) Richmond, Virginia (23298) USA; 5 Department of Biostatistics, Virginia Commonwealth University, (730 East Broad Street) Richmond, Virginia (23298) USA; 6 Department of Biochemistry and Molecular Biology, Virginia Commonwealth University, (1101 East Marshall Street) Richmond, Virginia (23298) USA

## Abstract

**Background:**

Clot strength by Thrombelastography (TEG) is associated with mortality during trauma and has been linked to severity of tissue hypoperfusion. However, the optimal method for monitoring this important relationship remains undefined. We hypothesize that oxygen transport measurements will be associated with clot strength during traumatic shock, and test this hypothesis using a swine model of controlled traumatic shock.

**Methods:**

N = 33 swine were subjected to femur fracture and hemorrhagic shock by controlled arterial bleeding to a predetermined level of oxygen debt measured by continuous indirect calorimetry. Hemodynamics, oxygen consumption, systemic central venous oxygenation (ScvO_2_), base excess, lactate, and clot maximal amplitude by TEG (TEG-MA) as clot strength were measured at baseline and again when oxygen debt = 80 ml/kg during shock. Oxygen transport and metabolic markers of tissue perfusion were then evaluated for significant associations with TEG-MA. Forward stepwise selection was then used to create regression models identifying the strongest associations between oxygen transport and TEG-MA independent of other known determinants of clot strength.

**Results:**

Multiple markers of tissue perfusion, oxygen transport, and TEG-MA were all significantly altered during shock compared to baseline measurements (p < 0.05). However, only ScvO_2 _demonstrated a strong bivariate association with TEG-MA measured during shock (R = 0.7, p < 0.001). ScvO_2 _measured during shock was also selected by forward stepwise selection as an important covariate in linear regression models of TEG-MA after adjusting for the covariates fibrinogen, pH, platelet count, and hematocrit (Whole model R^2 ^= 0.99, p ≤ 0.032).

**Conclusions:**

Among multiple measurements of oxygen transport, only ScvO_2 _was found to retain a significant association with TEG-MA during shock after adjusting for multiple covariates. ScvO_2 _should be further studied for its utility as a clinical marker of both tissue hypoxia and clot formation during traumatic shock.

## Background

Disordered hemostasis is present in up to 1/4 of severely injured trauma patients upon initial emergency department evaluation [1]. When present, it is associated with a four-fold increased mortality regardless of injury severity [1]. Clinical data and animal models have thus far, yielded strong evidence for a distinct biochemical aetiology for this early phemomenon that includes deregulated fibrinolysis and anticoagulation via the protein-C pathway that is linked to decreased vascular perfusion with tissue hypoxia [2-4].

Base deficit/excess has been used as the primary marker of tissue hypoxia used to predict early coagulopathy, mortality, and transfusion requirements in trauma patients [1,5-7]. In addition, blood lactate concentration is currently used to define the severity of hemorrhagic shock in animal models of trauma [4]. However, these metabolic markers of shock severity, while being readily clinical available, are not direct reflections of tissue hypoxia and can be affected by other factors during critical illness including liver/renal dysfunction and ethanol intoxication, thus limiting their utility [8-10].

Viscoelastic tests of clot formation such as Thrombelastography (TEG™) or Rotational Thrombelastometry (ROTEM™), have identified reduced clot strength, prolonged clot initiation times, and increased fibrinolysis in trauma patients [11-15]. Of these viscoelastic parameters, estimates of clot strength (maximal amplitude by TEG, and maximal clot firmness by ROTEM) are becoming increasingly favoured due to their good reproducibility and high sensitivity to the development coagulopathy and outcomes when compared to plasma-based assays [16,17]. Viscoelastic clot strength is an aggregate measurement that is dependent on multiple blood components including platelet activity and concentration, fibrinogen concentration, pH, hematocrit, and temperature [18-20]. It is this presence of multiple confounding influences on both markers of shock severity and viscoelastic clot strength that has made it difficult to precisely define how tissue perfusion is associated with changes in clot strength during trauma.

We have previously reported that clot strength by TEG is reduced in isolation prior to fluid resuscitation during traumatic shock in an oxygen debt-driven animal model [21]. This model affords a unique opportunity to examine the important relationships between changes in oxygen metabolism and clot strength during controlled traumatic shock in more detail. Better understanding of these relationships will inform further focused study on potential monitoring modalities and mechanisms of abnormal clot formation in the setting of traumatic shock.

In this study, we examine associations between oxygen transport/metabolism and clot strength by TEG in a swine model of controlled traumatic shock. We hypothesize that direct oxygen transport measurements will be associated with clot strength when measured during shock.

## Methods

### Swine Traumatic Shock Protocol

We used a Virginia Commonwealth University Institutional Animal Use Committee-approved swine model of anesthetized traumatic shock that was consistent with published international guidelines on the ethical treatment of animals. This model has been extensively described previously [21]. In brief, immature male swine weighing 40-50 kg were sedated with intramuscular ketamine/xylazine (20 and 2 mg/kg respectively) and surgical-plane anesthesia was induced with intravenous sodium pentathol (10-20 mg/kg). General anesthesia was then maintained using either intravenous alfaxalone (1 mg/kg bolus, 0.15 mg/kg/hr infusion) or alpha chloralose bolus (40-50 mg/kg bolus, 10 mg/kg/hr infusion). Of note, intravenous anesthesia was changed from alfaxalone to alpha-chloralose midway through the study due to difficulty obtaining a reliable supply of alfaxalone anaesthetic. Following induction of anesthesia, subjects were ventilated with room air (FiO_2 _= 21%) and respiratory rate was titrated to normalize PCO_2 _to 35-45 mmHg and was held constant for the remainder of the protocol. Subjects were also instrumented for continuous measurement of oxygen transport and hemodynamics and intermittent measurement of blood metabolism and coagulation during this period. After the brief baseline stabilization period, oxygen consumption (VO_2_) and mean arterial pressure (MAP) were recorded and a sample of whole blood was collected from the central venous circulation for blood gas, cell counts, and coagulation studies.

To add a component of tissue injury, soft tissue of both hind quarters was then traumatized and the right midshaft femur was fractured using a captive-bolt pistol causing an estimated Abbreviated Injury Scale (AIS) equal to 3 for the extremities [22]. Midline laparotomy was also performed using electrocautery and was assigned an estimated AIS = 2 yielding a total Injury Severity Score (ISS) equal to 13 [22].

Simultaneous with injury, the left femoral artery catheter was opened and blood was allowed to flow freely into a sealed graduated volumetric canister until MAP reached a predetermined goal of 30-35 mmHg. Hemorrhage was then halted and subjects maintained at goal MAP until oxygen debt (OD) accumulated to 80 ml/kg calculated by continuous indirect calorimetry at the airway. Goal MAP was maintained during the shock period by additional small blood draws or small aliquots (≤50 ml) of normal saline. Hemodynamic and oxygen transport measurements were recorded again and a second sample of whole blood for blood gas measurements, cell counts, and coagulation were obtained from the systemic central venous circulation at goal OD. No resuscitation was attempted during this time period and room air ventilation at the baseline rate was held constant. Upon completion of the protocol, subjects were euthanized by injection of potassium chloride (2 ml/kg) under anesthesia. Normal porcine body temperature (38° +/- 1 C) was maintained by a warming blanket and monitored continuously by rectal probe.

### Measurements

VO_2_, oxygen deficit, and OD were measured continuously breath by breath using indirect calorimetry at the airway at a frequency of 200 measurements per minute and were recorded using integrated software (BIOPAC Systems Inc., Goleta, CA). OD represents the total oxygen deficit accumulated over time during shock. OD starts at zero at baseline and increases in proportion to the magnitude and duration of oxygen deficit incurred during hemorrhagic shock. Previous work has demonstrated that OD is a sensitive marker of shock severity and a reliable predictor of mortality in similar swine models [23,24].

Blood gas analysis was made using the Stat Profile Critical Care Xpress bedside analyzer (Nova Biomedical Corp., Waltham, MA) to measure pH, base excess (BE), systemic central venous oxygen saturation (ScvO_2)_, and lactate concentration. The VetScan HM2 Hematology System, bedside analyzer (Abaxid, Union City, CA) was used to measure leukocyte count (WBC), hemoglobin concentration (Hgb), and platelet count (Plt). Blood for coagulation studies was collected into citrated vacutainers from a central venous catheter (Edwards Life Sciences, Irvine, CA) placed through the internal jugular vein to the right atrium as verified by pressure waveform. The START-4 coagulation analyzer (Diagnostica Stago, Asnières, France) was used to measure prothrombin time (PT) activated partial thromboplastin time (aPTT), and fibrinogen in platelet-poor plasma after centrifugation. TEG (TEG 5000, Haemoscope Corporation, Niles, IL) by recalcification (10 mmol/l final calcium concentration) was performed in whole blood according to manufacturer specifications at 37°C after 30 minutes and up to 3 hours after blood draw in all cases, which is a longer period than recommended by the manufacturer, but has demonstrated stability using citrated and recalcified samples [25]. TEG parameters measured included: clot onset time (R), clot formation (or kinetics) time (K), clotting angle (Angle), maximal clot strength (MA), and shear elastic modulus (G). All devices were calibrated as directed by the manufacturers.

### Variable Selection

Our overall goal was to examine the associations between changes in markers of oxygen transport and changes in viscoelastic clot strength. In order to do so, linear regression models were developed using TEG-MA as the primary outcome variable due to its sensitivity in identifying early functional coagulation changes compared to plasma-phase assays [17]. MA is an aggregate measure of clot strength and is influenced by blood pH, temperature, platelet count and activity, and fibrinogen concentration. No exact description of the relative contribution of each underlying factor to the overall development of MA exists, although, it is generally accepted that MA is primarily determined by platelet function and fibrinogen concentration [19,20]. The TEG "functional fibrinogen™" assay can isolate the fibrin contribution to MA using platelet inhibition. However, this assay was not included in our study because a similar thrombelastometry assay (FIBTEM™) was found not to be applicable to porcine blood [26]. We also included all known and measurable determinants of MA that were not standardized during the hemorrhage protocol. Therefore, the variables Plt, fibrinogen, pH, and Hct were considered as possible covariates when building the regression models due to their known influence on MA. These variables were included primarily to determine their role as covariates or confounders when evaluating the relationship between oxygen transport and clot strength, and will be referred to as making up the 'covariates' group for simplicity.

Direct measurements of VO_2_, ScvO_2_, BE and lactate were considered as the primary oxygen transport variables in the analysis. VO_2 _represents total body oxygen consumption and is calculated by the difference in absolute volume of inhaled and exhaled oxygen with each breath. BE represents the number of hypothetical base units required to return a sample of blood to neutral physiologic pH. Negative BE values during shock can represent tissue hypoperfusion with metabolic acidosis. ScvO_2 _represents the hemoglobin oxygen saturation in the central venous circulation and is determined by both the supply of oxygen to the tissues and the degree to which oxygen is extracted from the blood by the tissues. Lactate is a by-product of anaerobic metabolism and increases as mitochondrial oxygen supplies become limited and metabolism shifts to predominantly anaerobic glycolysis.

### Bivariate Analysis

The first step in selecting the appropriate variables for inclusion in the linear regression models was to determine the existence of strong bivariate relationships within each group of variables. This step identified any significant colinearity or interaction that might affect the final regression models. Oxygen transport and covariates were evaluated for 1^st ^order bivariate correlations among variables within each group. In addition, MA at baseline and MA at OD = 80 ml/kg were correlated in order to determine the influence of the baseline values on the values recorded during shock. Our primary interest is in the effect of the *change *in oxygen transport and its relationship to the *change *in clot strength. Therefore the difference (*Delta*) between each variable at baseline and during shock was also calculated and subjected to bivariate correlation within each group.

The second step in selecting variables for inclusion into linear regression models was to identify the predictor variables having the strongest bivariate relationship with MA. Therefore, each predictor's first- and second-order terms were related to both MA at OD = 80 ml/kg and *Delta *MA.

### Linear Regression

Predictor variables demonstrating moderate bivariate correlation (R > 0.4) with the value of MA measured during shock and *Delta *MA were considered for linear regression analysis. In the event of significant colinearity between oxygen transport variables, we planned to select the single representative oxygen transport variable with the strongest correlation with MA to include in the final regression analysis. This variable was then made available for selection as an independent variable during final model selection along with the described covariates and their interaction terms. Several linear regression models were then selected using forward stepwise variable selection with the absolute value of MA measured during shock and *Delta *MA as the two dependent outcomes. All statistical analysis was performed using JMP 8.0.1^® ^statistical software (SAS Institute Inc. Cary, NC).

## Results

A total of 33 swine weighing (Mean/std) 45.7(5.4) kg completed the traumatic hemorrhage protocol and achieved OD = 81.3(3) ml/kg after a period of 81.7(31.2) minutes in shock. Blood loss was 1089.2(319.3) ml, or 24 ml/kg, and animals received 85.7(184.6) ml of saline to maintain goal MAP during shock. Core temp was 37.9(0.6) deg C at the end of the shock period. Of these subjects, 52% (17/33) were anesthetized using alfaxalone anesthesia before changing to alpha chloralose. Paired T-test revealed no significant difference in MA recorded at baseline, during shock, or the change in MA between the two anesthetic regimens (p > 0.2). Consequently, the type of anesthesia was not included as a covariate in the final analysis.

Table 1 demonstrates the mean value of each oxygen transport, cell count, and coagulation variable recorded during the protocol at baseline and during shock. On average, all oxygen transport variables changed significantly from baseline to shock. In addition, average lactate increased to >6 mmol/L during shock indicating that a severe shock state was achieved. This level of lactate met previously used criteria for the development of coagulopathy in other animal models [4]. These changes were accompanied by a mild shift towards acidosis during shock that was significantly different from baseline values.

**Table 1:** Summary of oxygen transport, physiologic, and coagulation measurements.

	Baseline	Hemorrhagic Shock					
	
	Mean	Mean	Mean Diff	Std Err Diff	**95% CI Diff**.		p value
**Hemodynamics/Perfusion**							
MAP (mmHg)	110.1	29.7	-80.4	2.9	-86.7	-74.2	< 0.001
VO_2 _(ml/kg/min)	4.8	3.9	-1.1	0.1	-1.2	-0.9	< 0.001
ScvO_2 _(%)	65.1	18.4	-47.7	2.2	-52.1	-43.3	< 0.001
BE	1.8	-3.5	-5.6	0.7	-7.1	-4.2	< 0.001
Lactate (mmol/L)	1.5	7.1	+5.8	0.4	4.9	6.6	< 0.001
pH	7.42	7.35	-0.07	0.01	-0.09	-0.05	< 0.001

**Coagulation**							

PT (sec)	13.1	13.8	+0.7	0.3	0	1.4	0.048
aPTT (sec)	25.3	23	-2.3	1.7	-6.2	1.6	0.207
Fibrinogen (mg/dl)	154.5	82.8	-87.8	15.4	-121.5	-54.2	< 0.001

**Thrombelastography**							

R (min)	5.4	5.1	0.0	0.4	-0.8	0.8	1.0
K (min)	1.5	1.5	0.0	0.1	-0.2	0.2	1.0
Angle (°)	70.5	70.2	-0.8	1.9	-3.3	4.9	0.67
MA (mm)	68.7	65.2	-3.5	0.9	-5.3	-1.7	< 0.001
G (dynes/cm sqr.)	11275.2	9592.3	-1660.6	399.8	-2508.2	813.1	< 0.001

**Cell Counts**							

WBC (10^9/L)	13.9	14.5	-0.2	0.8	-1.9	1.5	0.83
Hgb (g/dl)	9.9	8.9	-1.0	0.2	-1.5	-0.5	< 0.001
Hct (%)	29.8	27	-2.7	0.6	-3.9	-1.5	< 0.001
Plt (10^9/L)	299.2	270.2	-24.7	10.6	-46.4	3	0.27

Data presented as mean, mean difference and standard error of the difference with 95% confidence intervals. Baseline measurements made prior to onset of hemorrhagic shock. Hemorrhagic shock measurements made after hemorrhage and a period of shock when Oxygen Debt = 80 ml/kg. All metabolic, coagulation, and cell counts measured from central venous blood samples. VO_2_= Total body oxygen consumption; ScvO_2_%= percent systemic central venous oxyhemoglobin saturation; BE = base excess of the extracellular fluid; PT = Prothrombin Time, aPTT = Activated Partial Thromboplastin Time; R = clot onset time, K = clot kinetics time, Angle = clotting angle, MA = clot maximal amplitude, G = clot shear modulus, WBC = white blood cell count; Hgb = hemoglobin concentration; Hct = percent hematocrit; Plt = Platelet Count

Hemoglobin, hematocrit, and platelet count were each decreased by 9-10% during shock compared to baseline measurements. (Table 1) This likely suggests a degree of auto resuscitation or mild dilution taking place during the hemorrhagic shock period which may have been amplified by continuous maintenance of hypotensive blood pressure by selective blood draws and normal saline titration [27].

Overall, coagulation parameters reflected no change in clot formation kinetics with a reduced, but not abnormal, MA in the setting of low fibrinogen. PT was slightly, but significantly, prolonged during shock when compared to baseline yielding a PT baseline/shock ratio of 1.05. In addition, aPTT was shortened but not significantly so, and fibrinogen fell significantly to approximately 54% of baseline values during shock. MA demonstrated a statistically significant 5% reduction during shock when compared to baseline values (68.7-65.2 mm, respectively) but did not become abnormal by standard definitions.

### Bivariate Analysis

Of the measured oxygen transport variables, significant colinearity was found only between the *Delta *BE and the *Delta *lactate during shock (R = -0.66, p < 0.001) and the absolute values of BE and lactate measured during shock (R = -0.7, p < 0.001). Of the covariates, significant colinearity was found between the *Delta *fibrinogen and the *Delta *pH (R = -0.59, p = 0.03). Among all other possible combinations, we found that fibrinogen and lactate measured during shock correlated negatively (R = -0.59, p = 0.03). Blood pH and VO_2 _measured during shock also correlated negatively (R = -0.80, p < 0.001). No other significant bivariate relationships between oxygen transport variables and covariates were found.

MA measured during shock was found to have a high-degree of positive correlation with baseline MA (R = 0.69, p = 0.002). Therefore, baseline MA was used as a covariate when identifying significant relationships between oxygen transport variables and the point measurement of MA during shock. This adjustment is necessary to avoid undue influence of variation in baseline MA between subjects. The same correction was not needed when examining the relationship between the predictor variables and the *Delta *MA for each subject. Of note, there was no bivariate association between volume of saline administered during shock and MA measured at OD = 80 ml/kg or the *Delta *MA (p > 0.2).

We then determined 1^st ^and 2^nd ^order associations of each predictor variable with MA measured during shock (adjusted for baseline MA) and the *Delta *MA. Of the oxygen transport predictor variables, only ScvO_2 _was found to have a significantly positive 2^nd ^order association with MA measured during shock after adjustment for baseline MA (overall model R^2^= 0.7, p < 0.001). In addition, ScvO2 measured during shock had a significant positive 2^nd ^order correlation with the *Delta *MA (R^2 ^= 0.69, p = 0.01).

### Multiple Linear Regressions

The ScvO_2 _2^nd ^order term was then used to represent oxygen transport in all models due to its strong bivariate relationship with MA. No colinearity was found between the value of ScvO_2 _measured during shock or the change in ScvO2 from baseline and other oxygen transport variables. Two multivariate models (Table 2) were selected using forward stepwise variable selection with a 0.25 probability to enter as follows:

**Table 2:** Selected linear regression models.

Independent Variable	F Ratio	p value	Outcome Variable	Whole Model R^2^	Whole Model p value
ScvO_2_	57.7	0.083	Clot Strength (MA) at OD = 80 ml/kg	0.99	0.032
MA (baseline)	513	0.028			
Fibrinogen	44.7	0.095			
Hematocrit	5.6	0.113			
ScvO_2_^2^	1.5	0.44			

ScvO_2_	508.5	0.028	*Delta *Clot Strength (MA)	0.99	0.029
Fibrinogen	830.6	0.022			
Platelet count	52	0.088			
ScvO_2_^2^	338.2	0.035			
(ScvO_2_*Platelet count)	141.9	0.053			

Summary of 2 linear regression models selected by forward stepwise variable selection per *Materials and Methods*. Each overall model was highly predictive of the MA measured during shock (OD = 80 ml/kg) or the change (*Delta*) in MA from baseline to shock. Fibrinogen and ScvO_2 _played important roles within each selected model.

1. The first regression model used the value of MA measured during shock as the dependent outcome variable. The covariates fibrinogen, pH, Hct, and Plt measured during shock and the 2^nd ^order ScvO2 term adjusted for baseline MA (y = β_0 _+ β_1_(ScvO_2_) + β_2_(MA at baseline) + β_3_(ScvO_2_^2^) were made available for selection as independent variables. Interaction terms between ScvO_2 _and each covariate were also made available for possible inclusion in the final model. With forward stepwise selection, fibrinogen and Hct measured during shock were added to the 2^nd ^order ScvO2 terms, making the final selected model highly predictive of MA during shock (R^2 ^= 0.99, p = 0.02). However, within the selected model there was no retained independent effect of the 2^nd ^order ScvO2 term on MA after adjusting for the added covariates.

$$\begin{array}{r} Equation:(MA\;at\;OD=80ml/kg)=\\ -41.3+0.37(ScvO_{2}\;at\;OD=80ml/kg)\\ +1.28(MA\;at\;baseline)\\ +0.05(fibrinogen\;at\;OD=80ml/kg)\\ +0.31(Hct\;at\;OD=80ml/kg)\\ +-0.004(ScvO_{2}^{2}) \end{array}$$

2. The second regression model utilized the *Delta *MA as the outcome variable. Again, the 2^nd ^order ScvO_2 _terms measured during shock were used as a starting point for forward variable selection. The same independent variables measured during shock with interaction terms were then added as possible covariates. The final selected model consisted of the ScvO_2 _second order term in addition to fibrinogen, Plt, and the interaction term (Plt*ScvO_2_). The overall model was highly predictive of the change in MA from baseline (Whole model R^2 ^= 0.99, p = 0.029). In this case, the 2^nd ^order ScvO_2 _term and fibrinogen each retained a significant effect on the *Delta *MA.

$$\begin{array}{r} Equation:(Delta\;MA)=\\ 33.2+-1.31(ScvO_{2}\;at\;OD=80ml/kg)\\ +-0.13(fibrinogen\;at\;OD=80ml/kg)\\ +0.001(Plt\;at\;OD=80ml/kg)\\ +0.05(ScvO_{2}^{2}\;at\;OD=80ml/kg)\\ +-0.005(ScvO_{2}*Plt\;at\;OD=80ml/kg) \end{array}$$

## Discussion

### Swine Model

The animal model satisfactorily produced a severe state of supply-dependent hemorrhagic shock by oxygen transport and metabolic markers which became significantly abnormal when OD = 80 ml/kg. However, the severe shock state combined with injury produced only an isolated reduction in MA without overt coagulopathy by standard definitions.

One reason for the lack of overt coagulopathy during shock may be our limited level of tissue injury. We calculated the total ISS = 13, which is less than that identified by Brohi et al, as being compatible with early coagulopathy [1]. However, the goal of the study was to isolate and examine the associations between tissue oxygen perfusion parameters and clot strength rather than to produce a significant overall coagulopathy. Increasing extremity injury would not have increased the ISS in our model per se. Thoracic injury would have likely confounded our oxygen debt measurements by impairing pulmonary oxygen exchange. Adding abdominal solid organ injury would have detracted from our ability to standardize shock severity due to uncontrolled hemorrhage. Inducing traumatic brain injury would have induced specific changes in clotting function, making interpretation of our results difficult. For these reasons, we limited ISS in order to better examine the specific associations between oxygen transport variables and TEG-MA.

Hypothermia was also prevented and plasma dilution was limited to that occurring from transcapillary refill and small aliquots of isotonic crystalloid during the hypotensive period. The 9-10% reduction noted in Hct and cell counts likely did not play a significant role in the measured significant decrease in MA from baseline. Small volume dilution of blood (less than 10% changes in Hct) with isotonic crystalloid has been shown *in vitro *to instead produce procoagulant properties to the blood and increase MA in healthy humans [28]. Overall, the animal model achieved the stated goal by providing an experimental platform in which significant changes in both oxygen transport and clot strength were achieved in the setting of traumatic shock, but should not be interpreted as producing an overt coagulopathy by current definitions.

Porcine models of coagulopathy in the setting of trauma are popular and favored because they use a large mammalian species that shares gross cardiovascular physiology with humans. Swine are amenable to precise monitoring while providing adequate sample volumes for viscoelastic testing. A review of experimental traumatic coagulopathy models found that of 33 models deemed appropriate for review, 17 were porcine [29]. However, significant differences exist in the type of coagulation changes produced in swine in response to hemorrhage and these differences are important to consider when interpreting our results.

Standard tests of blood coagulation function typically demonstrate pro-coagulant activity in swine compared to humans, and immunologic methods are not generally comparable as illustrated in a comparison of 22 commercial assays in healthy pigs and humans by Munster et al [30]. The authors found that PT was approximately equal between species while aPTT was shorter in pigs suggesting enhanced intrinsic coagulation cascade activity. In addition, plasma tissue factor levels were 4-fold higher in pigs, which may have special relevance in the setting of trauma since coagulopathic trauma patients have demonstrated increased plasma tissue factor activity [31]. Using ROTEM, comparisons of porcine and human clot formation also suggest a hypercoagulable state in pigs relative to humans. Pigs tend to demonstrate shorter clot formation times, faster clot buildup, and increased maximal clot firmness with similar clot lysis profiles to humans [26,32]. TEG parameters correlate with ROTEM in porcine blood, with TEG demonstrating higher values for clotting angle and clot strength (MA vs. MCF) [33]. Therefore, the native hypercoagulable state of porcine blood relative to humans may require that a greater degree of shock or increased injury severity be incurred in order to accurately reproduce the early coagulation changes seen in humans. This species difference may have contributed to our lack of overt coagulopathy during shock.

To date, no porcine model has accurately reproduced the initial hemostatic changes observed in human traumatic coagulopathy. Sapsford et al, observed no change in PT after 40% hemorrhage compared to baseline measurements using an aortic tear model [34]. Martini et al, observed no difference in PT, R, K, Angle, and a significant, but limited, reduction in MA (approx 67 to 63 mm) measured 4 hours after 35% hemorrhage combined with crystalloid resuscitation of 3 times shed blood volume [17,35]. Via et al, reported in their sham resuscitation group no change in PT, PTT, or fibrinogen, and a reduction in TEG-MA from 74-71 mm at one hour of shock after a 40% blood volume hemorrhage [36]. Using ROTEM, Haas et al, reported that clotting time and clot formation time were essentially unchanged and maximal clot firmness was reduced, but not necessarily abnormal, after a 60% blood volume hemorrhage [37]. Cho et al, reported a multi-institute porcine model that, similar to ours, added femur fracture by captive-bolt pistol [38]. When compared to our model, they achieved a similar injury profile, hemorrhage volume, and a similar level of lactate accumulation during shock. Their coagulation parameters measured at "End of Shock" after injury and hemorrhage, but prior to fluid resuscitation, are most likely comparable to our OD = 80 ml/kg measurements. At this particular time point, they found an INR baseline/shock ratio of only 1.1 and TEG parameters demonstrating a trend towards hypercoagulability (R, K, and Angle) with an isolated decrease in MA that was not outside the baseline reference range [38]. Overall, the available viscoelastic porcine data demonstrates a tendency for isolated and limited decrease in clot strength as the initial response to hemorrhage. This result agrees with our own and is somewhat dissimilar to human observational studies which typically demonstrate a mixed impairment of prolonged clot onset times and decreased clot strength. This initial response may be species-specific. Alternatively, current porcine models may lack the appropriate criteria (combined shock and injury severity) to induce very early coagulopathy similar to that seen in humans. Our model is also limited in this respect since we achieved only and ISS = 13. Therefore, our results, while consistent with other porcine models, may not be directly comparable to traumatic coagulopathy observed in human studies.

### Fibrinogen Consumption

Fibrinogen was rapidly consumed during shock, consistent with previously published results using similar swine models. This likely reflects an increased consumptive process associated with the injury and shock state since acidosis was minimal [39,40]. Systemic venous pH and lactate both correlated with fibrinogen during shock. Direct acidification of the blood can reduce circulating fibrinogen levels by increasing breakdown without increasing production [40]. However, the underlying mechanism of this effect of pH on fibrinogen metabolism remains unknown. In addition, the lack of a direct association of oxygen consumption with fibrinogen and the mild overall acidosis indicates that the reduction in fibrinogen we observed should not be attributed entirely to the effects of tissue hypoperfusion or acidosis. Alternatively, the rapid consumption of fibrinogen may be attributable to the chosen pattern of injury since femur fracture and femur fracture manipulation have been associated with rapid consumption of fibrinogen in both animal models and human studies [41,42].

### Oxygen Transport and Clot Strength

Forward variable stepwise selection revealed that ScvO_2_, fibrinogen, Hct, and platelet count were important predictors of clot strength in this animal model. There was also evidence for an interaction between ScvO2 and platelet count in determining *Delta *MA during shock suggesting a specific role for platelets. Each selected linear regression model was highly predictive of both the value of MA during shock and *Delta *MA from baseline. The lack of a direct association between VO_2 _and clot strength and the importance of ScvO_2 _as the only significant oxygen transport associated with MA was interesting and surprising. This finding was even more surprising when considering that BE and lactate, the current metabolic markers used clinically to define tissue hypoperfusion, shared no association with clot strength in our animal model. Lactate did correlate with fibrinogen concentration during shock, but was not directly associated with MA. Therefore, it is possible that fibrinogen may have confounded an underlying association between lactate and clot strength. Alternatively, another physiologic variable (such as acidosis) mediates this relationship, but was not sufficiently pronounced in our model.

The reason why ScvO_2 _was more strongly associated with clot strength when compared to other direct markers of oxygen transport or tissue hypoperfusion remains unclear. One explanation is that lactate produced in hypoperfused tissues may not have reached the central circulation by "wash out" prior to reperfusion, thus lactate may be less accurate than ScvO_2 _in terms of hypoperfusion prior to fluid resuscitation. Among hemodynamic and oxygen transport measurements, ScvO2 has been found by Scalea et al, to be the best predictor of acute blood loss in experimental trauma models [43]. The authors suggest that this sensitivity is a result of the ability of ScvO2 to reflect early increasing oxygen extraction at the blood/tissue interface in response to hemorrhage before gross hemodynamic measurements become abnormal.

In our study, the same sensitivity of ScvO2 to early changes in oxygen extraction may potentially explain its strong association with clot strength via compensatory endothelial activation in response to hypoxia. The observed fall in ScvO_2 _and VO_2 _with a concurrent increase in lactate confirms that oxygen delivery to the tissues was reduced below critical levels, despite maximal oxygen extraction. In addition, the disproportionately large fall in ScvO_2 _from baseline levels (reduced 72%) when compared to VO2 (reduced 19%) suggests that blood oxygen extraction was actively enhanced at the blood/endothelial interface during shock. Therefore, we speculate that ScvO_2 _and clot strength may be associated via activation of the endothelium as part of the local endothelial response to hypoxic conditions [44]. While we did not directly measure biomarkers of endothelial activation, further evidence for a link between ScvO_2_, protein C, and endothelial activation was recently reported by Trecziak et al. in critically ill septic patients. The authors used ScvO_2 _to measure hypoxia and its effect on coagulation measurements and found that a subgroup of patients with both abnormally low ScvO_2 _plus hypotension demonstrated changes in protein C, thrombomodulin, and increased endothelial activation by E-selectin expression [45]. Therefore, our findings taken in this context may indirectly support the mechanism put forth by Brohi et al., who described a critical role for endothelial activation of protein C in the pathophysiology of trauma-induced coagulopathy [2]. Future research on this topic should seek to include biomarkers of endothelial activation when examining associations between tissue hypoxia/hypoperfusion and clot formation.

### Limitations

We acknowledge that there are distinct limitations to this study. As discussed, the relevance of the swine model to human subjects is concerning due to the native differences between porcine and human coagulation function. In addition, we calculated the coefficient of variation (CV) for swine MA measured at baseline in the study of Cho et al., and found it to range from 12-20% across centers [38]. Our 5% change in MA from baseline to shock is well within this range, further limiting our results. In addition, tissue injury was limited and the model itself achieved only a mild reduction in clot strength without overt coagulopathy. We also did not strictly standardize the timing of TEG test performance, possibly adding variability to our results. However, when taken in the context of other similar swine models of hemorrhage, the changes in clot strength in our model were quite similar to those described by other investigators when measured during shock and prior to fluid resuscitation.

We intended to isolate the association between oxygen metabolism and clot strength so to examine the inherent relationships in detail. As a result, we can only speculate on the associations found between independent and dependent variables and cannot make any causative or mechanistic conclusions from the data. Nevertheless, the associations found suggest important areas for further focused study concerning the early detection and monitoring of hemostasis during trauma.

## Conclusions

In summary, ScvO_2 _was associated with reduced clot strength by TEG during traumatic shock in this swine model of controlled hemorrhage. Fibrinogen, hematocrit, and platelet counts were found to be important covariates in this relationship. These findings suggest that, perhaps due to its association with tissue oxygen extraction, ScvO2 deserves further study as a potentially useful clinical marker of both tissue perfusion and clot formation during trauma.

## Abbreviations

AIS: Abbreviated Injury Scale; aPTT: Activated Partial Thromboplastin Time; BE: Base Excess; *Delta *: Difference; Hgb: Hemoglobin; ISS: Injury Severity Score; MA: Maximal Amplitude; MAP: Mean Arterial Pressure; OD: Oxygen Debt; PCO_2 _: Partial Pressure of Carbon Dioxide; Plt: Platelet Count; PT: Prothrombin Time; ROTEM: Rotational Thrombelastometry; ScvO_2: _Systemic Central Venous Oxygen Saturation; TEG: Thrombelastography; TIC: Trauma Induced Coagulopathy; VO_2 _: Total Body Oxygen Consumption; WBC: White Blood Cell Count

## Competing interests

The authors declare that they have no competing interests.

## Authors' contributions

NJW and EJM participated in sample collection and coagulation testing. NJW, YS, and DFB participated in study design, developing appropriate statistical methods, and data analysis. NJW, KRW, and RFD participated in design and management of the traumatic shock animal model. All authors contributed to the study coordination and helped to draft the manuscript. All authors read and approved the final manuscript.
